# Management of Diabetic Ketoacidosis with Dengue Hemorrhagic Fever

**DOI:** 10.7759/cureus.3336

**Published:** 2018-09-19

**Authors:** Khemaporn Lertdetkajorn, Chutintorn Sriphrapradang

**Affiliations:** 1 Medicine, Ramathibodi Hospital, Mahidol University, Bangkok, THA

**Keywords:** capillary leak syndrome, diabetes complications, fluid therapy, severe dengue, viral hemorrhagic fever

## Abstract

Viral hemorrhagic fever is one of the most important emerging infectious diseases. Some viral hemorrhagic fevers include dengue, hantavirus, Ebola infection, and yellow fever. Dengue virus infection results in a wide spectrum of clinical diseases, including dengue hemorrhagic fever, characterized by the transient period of plasma leakage and hemorrhagic tendency. Vascular instability ranges from mild to fatal shock, and hemorrhage varies from none to life-threatening. Infection is the predominant precipitating factor for diabetic ketoacidosis. In addition to insulin administration, successful management of diabetic ketoacidosis requires fluid resuscitation. We herein report an adult patient with diabetic ketoacidosis complicated with dengue hemorrhagic fever who developed leakage syndrome. Early recognition of leakage and appropriate fluid management was critical in the diabetic ketoacidosis management of this case.

## Introduction

Viral hemorrhagic fevers (VHFs) are a group of systemic viral infections, including Ebola, Marburg hemorrhagic fever, Lassa fever, Rift Valley fever, Crimean-Congo hemorrhagic fever, hantaviruses, yellow fever, and dengue. VHFs can lead to a wide range of clinical manifestations, from minor bleeding to fatal hemorrhagic fever characterized by vascular leakage and bleeding, which may progress to multiple organ failure.

Dengue has been known as one of the world's most common and rapidly spreading mosquito-borne viral diseases. Dengue has become endemic in the Caribbean, South America, and Africa, in addition to well-recognized endemic areas in Southeast Asia. It has the potential to become an endemic disease in the new areas that were not previously affected [1]. Dengue is predominantly a pediatric disease, but there has been an increasing incidence reported in adults over the past decades [2]. 

Infection is one of the most prevalent precipitating causes of diabetic ketoacidosis (DKA). Major components in the treatment of DKA include fluid replacement therapy, insulin administration, and correction of electrolyte abnormalities. Vigorous fluid replacement using isotonic saline is the critical initial step in the management of DKA. In contrast, it is important to avoid unnecessary fluid therapy with the goal of minimizing the possibility of fluid leakage into the third space in the patient with dengue hemorrhagic fever (DHF) [3]. We present a case report that highlights the careful fluid management of DKA in a patient with DHF. The abstract was presented at the ENDO2018 in Chicago, IL, USA on April 17-20, 2018 (http://www.endocrine.org/meetings/endo-annual-meetings/abstract-details?ID=5315).

## Case presentation

A 51-year-old woman with no prior underlying disease, presented with four days of fever, myalgia, and vomiting. Previously, she had a history of polydipsia, polyuria, nocturia, and weight loss for one month. She denied tobacco, alcohol, or illicit drug use. There was no family history of diabetes mellitus. On examination, her blood pressure was 115/78 mm Hg, the temperature was 36.6^o^C, and pulse rate was 96 beats per min. Volume status was mildly dehydrated. Venous plasma glucose was recorded at 467 mg/dL. Wide anion gap metabolic acidosis and high levels of serum beta-hydroxybutyrate were present. A diagnosis of DKA was made based on the above findings. Clinical features and laboratory investigations are summarized in Table 1. An electrocardiogram showed sinus tachycardia and a chest X-ray showed no abnormalities. She was initially treated with isotonic saline of 1 liter (L) in one hour. Subsequently, intravenous isotonic saline was continuously administered at the rate of 500 mL/hr, along with the repletion of potassium. A bolus of insulin, followed by continuous insulin infusion, was administered.

**Table 1:** Summary of Clinical Features and Laboratory Investigations in This Patient CBC: complete blood count; HbA1c: hemoglobin A1c; HCO_3_: bicarbonate; IgG: immunoglobulin G; IgM: immunoglobulin M; Na: sodium; NS1: non-structure protein 1; pH: potential of hydrogen; WBC: white blood count

Diagnosis	Clinical Manifestations	Laboratory Results
Diabetic ketoacidosis	Polydipsia, polyuria, and nocturia for one month; mild dehydration	Venous plasma glucose 467 mg/dL; HbA1c 13.35%; beta-hydroxybutyrate 5.8 mmol/L; venous pH 7.39; Na 125, HCO_3_ 13 mmol/L; calculated anion gap 18 mmol/L; urine specific gravity 1.032
Dengue hemorrhagic fever	Acute high-grade fever for four days, myalgia, vomiting, vaginal bleeding	CBC: hematocrit 48%; platelet count 17,000 /mm3; WBC 8,800 /mcL with 60% neutrophils, 16% lymphocytes, 10% monocytes, and 13% atypical lymphocytes; Dengue virus NS1 antigen, Dengue IgG and IgM: all positive

**Figure 1:**
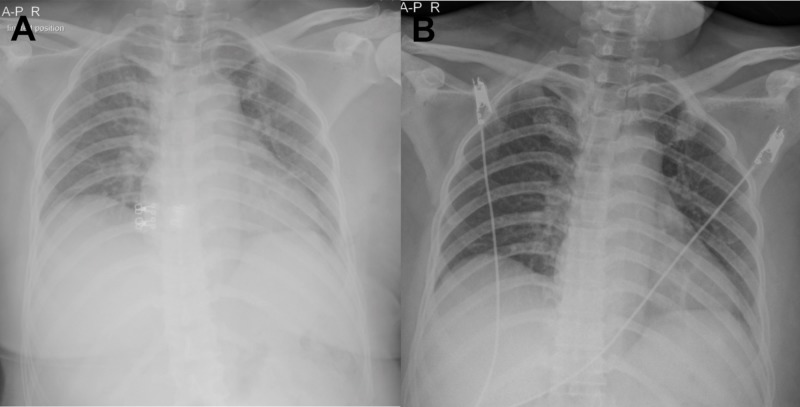
Chest X-rays During Treatment Course A) Chest x-ray showed bilateral hilar congestion six hours after aggressive fluid therapy during the plasma leakage stage of dengue hemorrhagic fever; B) Resolution of pulmonary congestion following the appropriate reduction of intravenous fluid.

In addition, the etiology of her acute fever was revealed as dengue infection (laboratory results in Table 1). A systemic review found that she currently had vaginal bleeding. DHF was evidenced by hemoconcentration, thrombocytopenia, and hemorrhagic manifestation. After six hours of treatment, she complained of chest discomfort and developed puffy eyelids and mild pitting edema in both legs. Lung examination revealed fine crepitation in both lungs. A chest x-ray revealed bilateral hilar congestion (Figure 1A). These abnormalities occurred after the administration of 3 L of isotonic saline. After the detection of leakage syndrome, the rate of intravenous fluid was decreased from 140 to 60 mL/hr. Urine output was closely monitored every hour. The fluid management was aimed at a negative balance. The patient’s clinical status gradually improved and repeated chest x-ray demonstrated no sign of fluid leakage (Figure 1B). Eight hours after being admitted, there was finally a resolution of the DKA in the patient without additional complications. The patient was discharged the fifth day of admission with a premixed insulin injection. At her eight-month follow-up, she was able to discontinue insulin therapy and received a triple combination of oral hypoglycemic agents as long-term therapy.

## Discussion

Symptomatic dengue infections were classified according to the World Health Organization (WHO) 1997 guidelines into dengue fever, DHF, and dengue shock syndrome [4]. The more recent WHO 2009 guidelines classified dengue according to levels of severity, which included dengue without warning signs, dengue with warning signs, and severe dengue [5]. However, the 1997 classification is still used in Thailand due to the high incidence of DHF and dengue shock syndrome. For this patient, DHF was diagnosed from the history of acute fever for four days, thrombocytopenia, hemoconcentration, and vaginal bleeding. Dengue shock syndrome is DHF accompanied by signs of circulatory failure. Vascular leakage is caused by a transient increase in vascular permeability due to endothelial dysfunction [6]. Hematocrit elevation of greater than 20% is typically used as a cut-off to define the presence of leakage in dengue. In patients with vascular leakage, intravenous fluid therapy can aggravate fluid accumulation and can lead to respiratory distress. Frequent monitoring and judicious use of intravenous fluids are essential in the management of patients with DHF. The purpose of initial fluid therapy is to administer the minimum intravenous fluid volume of isotonic solutions required to maintain good perfusion and urine output of about 0.5 ml/kg/hr. Intravenous fluids should be reduced gradually when the rate of plasma leakage decreases towards the end of the leakage period which is indicated by adequate urine output or hematocrit decreasing below the baseline [5]. Thus, it is necessary to revise the fluid infusion frequently.

DHF was considered as a trigger factor of DKA in this patient. To date, there have been only a few case reports of dengue infection precipitating DKA [7-8]. However, we believe that the rarity of case reports may underestimate the true incidence of DKA aggravated by dengue infection in our country. The osmotic diuresis in DKA results in large volume depletion. Typical total body water deficit among patients with DKA is 100 ml/kg of body weight, which is equivalent to 6 L [9]. In the absence of cardiac abnormalities, isotonic saline (0.9% saline) is infused at a rate of 500 - 1,000 mL/hr during the first two to four hours, followed by the infusion of 0.9% saline at 250 - 500 mL/hr or 0.45% saline, depending of the serum sodium level and the dehydration status [10]. Thus, intravenous fluid should be meticulously monitored during the leakage stage of DHF. Signs and symptoms of excess volume must be closely observed. The fluid management was aimed at a negative balance to prevent further complications, such as pulmonary edema and respiratory failure (which is the most severe complication that usually occurs from unnecessary hydration during the leakage stage).

In addition to DHF, the fluid management must be given more cautiously in the patient with underlying cardiac or renal disease. Special populations, such as the pediatric population, need gradual replacement of sodium and water deficits to prevent cerebral edema, which is an uncommon but potentially devastating complication. The pathophysiology of cerebral edema is not fully understood. The possible explanations could be from increased blood-brain barrier permeability, rapid osmotic changes, and fluid shifts that occur during DKA treatment [11].

## Conclusions

Special considerations should be given to cases of DKA occurring with DHF. These patients prone to have a leakage syndrome; therefore, caution should be exercised during volume resuscitation. Meticulous and frequent monitoring is an important step in taking care of these patients.
